# Mortality Associated with Recurrent Extreme Hyperferritinemia in Critically Ill Adolescents

**DOI:** 10.1155/2022/6207417

**Published:** 2022-03-22

**Authors:** John Scott Baird

**Affiliations:** Department of Pediatrics, Columbia University Vagelos College of Physicians & Surgeons, New York, NY, USA

## Abstract

**Introduction:**

Recurrent extreme hyperferritinemia (ferritin >10,000 ng/mL) was noted in 4 critically ill adolescents prior to death, though this association has not previously been described.

**Methods:**

A retrospective review of the medical records of 4 critically ill adolescents with recurrent extreme hyperferritinemia and systemic inflammation was performed to identify additional common epidemiologic factors.

**Results:**

Systemic inflammation was characterized as cytokine storm syndrome in 2 patients and hemophagocytic lymphohistiocytosis in 2 patients. Episodes of extreme hyperferritinemia were noted on at least 2 different dates in all patients; these episodes (*n* = 10) were separated by an interval of 2 weeks to several months and were usually (in 8 of 10 episodes) associated with the onset or worsening of multiple organ dysfunction syndrome. Death occurred within 2 weeks of the onset of an episode of recurrent extreme hyperferritinemia. Lymphocytopenia and cachexia were noted in all patients.

**Conclusions:**

Recurrent extreme hyperferritinemia—often with multiple organ dysfunction syndrome—was noted in 4 adolescents with systemic inflammation who did not survive their critical illness. Recurrent extreme hyperferritinemia may be a novel biomarker of increased mortality in patients with the syndrome of persistent inflammation, immunosuppression, and catabolism.

## 1. Introduction

Hyperferritinemia with a serum ferritin >3,000 ng/mL has been associated with an increased mortality compared to less-severe hyperferritinemia in critically ill pediatric patients: 26 of 68 (38%) PICU patients with serum ferritin >3,000 ng/mL did not survive hospitalization [1]. It is possible that mortality is increased further with an episode of extreme hyperferritinemia [2] (serum ferritin >10,000 ng/mL), though data are limited. Extreme hyperferritinemia in pediatric patients has been linked to hemophagocytic lymphohistiocytosis [3] (HLH), a disorder characterized by systemic inflammation, and 4 of 9 (44%) pediatric patients with HLH and extreme hyperferritinemia in that study did not survive [3].

The risks associated with recurrent hyperferritinemia are unknown, though it could be associated with an increased mortality. It is also possible that the magnitude of hyperferritinemia during recurrences contributes to the mortality risk.

This case series reviews the clinical course of 4 critically ill adolescents, each with more than 1 episode of extreme hyperferritinemia: none of them survived. Speculation regarding the significance of this pattern of association as a possible manifestation of the recently recognized persistent inflammation, immunosuppression, and catabolism syndrome (PICS) [4] is presented.

## 2. Methods

This case series was exempted from IRB approval by the Columbia University IRB (AAAT8703).

A retrospective review of the clinical course of 4 adolescents with recurrent extreme hyperferritinemia who died in the PICU of Morgan Stanley Children's Hospital of New York-Presbyterian from July 2017 through July 2021 was performed, including a review of relevant medical history and laboratory findings. All serum ferritin levels (hereafter, ferritin) as well as serum interleukin-6 levels (hereafter, IL-6) during each patient's hospitalization were reviewed. Our hospital's normal range for ferritin is 30 to 400 ng/mL (upper limit: 100,000 ng/mL), and the normal range for IL-6 is < 7 pg/mL. Additional serum tests reviewed in this study include C-reactive protein (CRP; normal range ≤10 mg/L; upper limit: 300 mg/L), d-dimer (normal range ≤0.8 microg/mL; upper limit: 20 microg/mL), IL-2 (normal range <1.9 pg/mL), and soluble IL-2 receptor (normal range: 622 to 1,619 pg/mL; upper limit: 20,000 pg/mL).

Cachexia as an indicator of a patient's nutritional state was defined (using newly proposed adult criteria [5]) as a weight loss of at least 5% in 12 months or less in the presence of 3 of the following 5 criteria: decreased muscle strength, fatigue, anorexia, low fat-free mass index, and specific laboratory findings (elevated CRP and IL-6 with anemia and hypoalbuminemia) [5]. Cytokine storm syndrome (CSS) was considered similar to cytokine storm [6] and diagnosed in patients without concurrent HLH but with hypercytokinemia and systemic symptoms (any of the following: fever, fatigue, anorexia, headache, rash, diarrhea, arthralgia, myalgia, or neuropsychiatric findings). HLH was defined by the presence of at least 5 of the following 8 criteria: fever, splenomegaly, bicytopenia, hypertriglyceridemia and/or hypofibrinogenemia, hemophagocytosis, low/absent NK cell activity, hyperferritinemia, and elevated soluble interleukin-2-receptor levels [7]. Immunosuppression was considered present in patients with persistent lymphocytopenia (an absolute lymphocyte count <1,000 per microL of blood) or neutropenia (an absolute neutrophil count (ANC) <1,000 per microL of blood). Multiorgan dysfunction syndrome (MODS) was defined as the dysfunction of at least 2 organ systems using PELOD criteria [8].

Statistical correlations were assessed by Spearman tests. Statistical tests were two-sided, with significance set at *p* < 0.05. All statistical calculations were obtained using Prism 9 (GraphPad Software, Inc.; San Diego, CA).

## 3. Results

These 4 patients all had systemic inflammation (2 with CSS, 2 with HLH) with an underlying disease (Table 1), and all were immunosuppressed (with lymphocytopenia) with a potentially life-threatening infection, including coronavirus disease 2019 (COVID-19) in 2 patients. They were all cachectic.

Patient #1 had the fewest values for ferritin (*n* = 11; vs *n* = 56, 42, and 42 values in patients #2, 3, and 4, respectively). Extreme hyperferritinemia was noted on at least two different dates in all patients; these episodes of extreme hyperferritinemia (*n* = 10) were separated by an interval of 2 weeks to several months, and each episode included from 1 to 17 consecutive values. Eight of 10 episodes of extreme hyperferritinemia were associated with the onset or worsening of MODS during the episode of extreme hyperferritinemia. Death occurred within 2 weeks of an episode of recurrent extreme hyperferritinemia.

Few paired values for IL-6 and ferritin were available for 3 patients (9, 4, and 3 paired values for patients #1, 2, and 4, respectively), while patient #3 had 36 paired values. IL-6 was elevated for each patient during at least 1 episode of extreme hyperferritinemia. Few paired values for CRP and ferritin were available for 3 patients (7, 2, and 0 paired values for patients #1, 2, and 3, respectively), while patient #4 had 33 paired values. Few paired values for d-dimer and ferritin were available for 3 patients (1, 2, and 9 paired values for patients #1, 2, and 4, respectively), while patient #3 had 24 paired values. Table 1 includes peak values for IL-6, CRP, and d-dimer for each patient. There were few levels of IL-2 (*n* = 3 in patient #2) or soluble IL-2 receptor (*n* = 3 in patient #2; *n* = 1 in patient #3) available for review.

While 3 patients were neutropenic during episodes of extreme hyperferritinemia (as well as during most of the remainder of their hospitalization), patient #2 was not.

### 3.1. Patient #1

**Figure 1:**
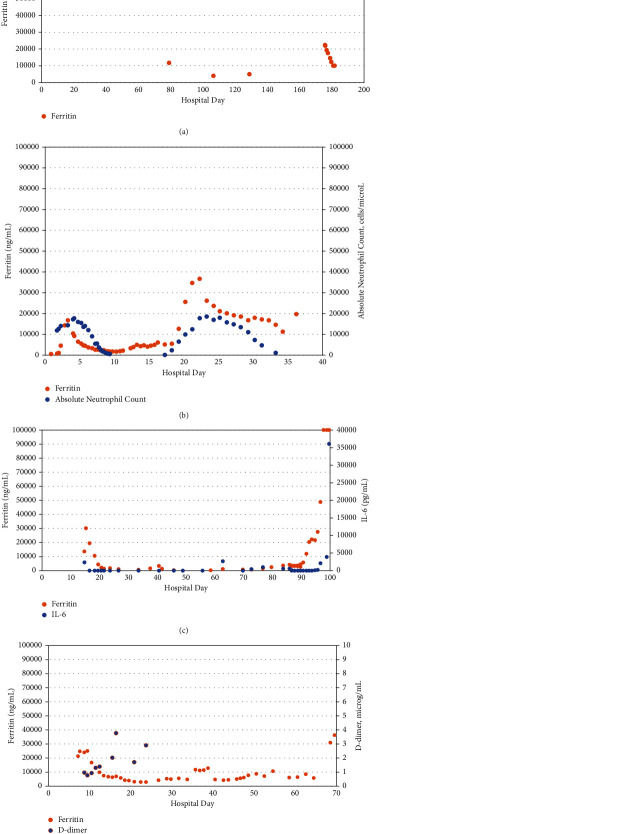
The time course of ferritin in 4 critically ill adolescents: (a) patient #1, (b) patient #2 (with the time course of the peripheral blood ANC), (c) patient #3 (with the time course of IL-6), and (d) patient #4 (with the time course of d-dimer).

A 15-year-old female with refractory aplastic anemia was evaluated for possible HSCT but was found to have COVID-19 infection. Her subsequent hospitalization lasted 6 months, and she was pancytopenic (with both lymphocytopenia and neutropenia) for most of her hospitalization; she was also cachectic. After 2 months in the hospital, her COVID-19 infection had resolved and she was treated with a conditioning regimen followed by a HSCT. Within a few days, her HSCT was complicated by pneumonia and typhlitis and the first ferritin level was elevated above 10,000 ng/mL on hospital day (HD) 79 (Figure 1(a)). Concurrently, IL-6 was also elevated (21.2 pg/mL). Over the next 3 months, she was transferred several times to the PICU with MODS; ferritin levels are not available for those events. Associated complications included methemoglobinemia, *Aspergillus* pneumonia, thrombotic microangiopathy, idiopathic pneumonia syndrome, and CSS (treated with tocilizumab, anakinra, and siltuximab).

Recurrent extreme hyperferritinemia was noted 1 week prior to her death (and 99 days following the first ferritin peak) concurrent with the onset of MODS and an increased IL-6 (510.9 pg/mL; she was treated with tocilizumab on HD 176, and IL-6 on HD 178 was 1,423 pg/mL). Her death on HD 182 was associated with pulmonary hemorrhage.

There was no autopsy for this patient.

In summary, 1 of the 2 episodes of extreme hyperferritinemia in this patient was associated with the onset of MODS. There was no association between the trends in ferritin and IL-6 (*p*=0.24) or CRP (*p*=0.66). There were too few paired values of d-dimer and ferritin to investigate an association.

### 3.2. Patient #2

A 16-year-old male with a history of Crohn's disease was admitted with fever, thrombocytopenia, and mildly elevated ferritin (478 ng/mL). A day after admission, he was in shock and was treated with vasopressors. MODS and extreme hyperferritinemia were noted on HD 3 (Figure 1(b)), and on HD 4, IL-6 was elevated (81 pg/mL) as was soluble IL-2 receptor (8,043 pg/mL), with a normal value for IL-2. HLH secondary to an occult malignancy was suspected, and treatment was initiated concurrently with diagnostic efforts: he received basiliximab (on HDs 5, 6, 17, and 18), corticosteroid, cyclophosphamide, etoposide, and tocilizumab (on HDs 4, 17, and 35). By the end of the first week, he developed persistent lymphocytopenia. *Candida albicans* was found on bronchoscopy on HD 10 and treated. IL-2 and IL-6 were both elevated (10.9 and >4,200 pg/mL, respectively; the last dose of tocilizumab was 9 days earlier) on HD 13, as was soluble IL-2 receptor (18,277 pg/mL). HLH was confirmed on HD 20. By the end of his third week in the hospital, he was cachectic in spite of continued parenteral nutrition.

Extreme hyperferritinemia recurred on HD 22 a few hours prior to the resection of a subxiphoid mediastinal mass (later diagnosed as histiocytic sarcoma) and worsening MODS. On HD 24, IL-2 and IL-6 remained elevated, though less so than earlier (3.2 and 448.6 pg/mL, respectively), while soluble IL-2 receptor was >20,000 pg/mL. Ferritin gradually declined over the next 12 days to a level close to 10,000 ng/mL. On HD 35, he was again in shock, and ferritin rose again on HD 36 coincident with an elevated IL-6 (>4,200 pg/mL; the last dose of tocilizumab was 18 days earlier), *Staphylococcus aureus* septic shock, and death.

An autopsy revealed disseminated histiocytic sarcoma in the colon, liver, lungs, lymph nodes, pancreas, and spleen, as well as *Candida* pneumonia and *Staphylococcus aureus* bacteremia.

In summary, both of the 2 episodes of extreme hyperferritinemia in this patient were associated with the onset or worsening of MODS. There were too few paired values of ferritin with IL-6 or CRP to investigate an association. There was no association between the trends in ferritin and d-dimer (*p*=0.09). The trend in ferritin correlated with the trend in peripheral blood ANC (*p*=0.00015).

### 3.3. Patient #3

A 16-year-old male with X-linked inhibitor of apoptosis deficiency associated with inflammatory bowel disease and multiple medical problems was admitted for a HSCT. He was cachectic with persistent pancytopenia (including both lymphocytopenia and neutropenia) during this hospitalization. Extreme hyperferritinemia was noted on HD 14 (Figure 1(c)) concurrently with the onset of MODS; hypercytokinemia (IL-6 was 2,369 pg/mL) was presumed secondary to HLH. He was diagnosed with COVID-19 infection on HD 18 and treated with tocilizumab for the first time. He was treated with tocilizumab again on HD 48 and again on HDs 62 and 63 when he underwent a HSCT; unfortunately, the HSCT was complicated by septicemia associated with *Klebsiella pneumoniae*, *Streptococcus mitis*, and *Enterobacter cloacae*. Over the next few weeks, he had an episode of anaphylaxis and developed sinusoidal obstructive syndrome.

He met criteria for HLH on HD 91 when he had recurrent extreme hyperferritinemia (which continued until his death) and was then treated with tocilizumab. On HD 95, MODS recurred; he was treated with anakinra on HD 97; soluble IL-2 receptor was elevated (5,143.4 pg/mL) on HD 98; and on HD 99, IL-6 was elevated (36,115 pg/mL; the last dose of tocilizumab was 8 days earlier), and he died later that day.

An autopsy noted *Stenotrophomonas maltophilia* in the blood and lungs without an inflammatory response in the lungs.

In summary, both of the 2 episodes of extreme hyperferritinemia in this patient were associated with the onset of MODS. The trend in ferritin correlated with the trend in IL-6 (*p*=0.047). There were no paired values of CRP and ferritin, and there was no association between the trends in ferritin and d-dimer (*p*=0.09).

### 3.4. Patient #4

A 19-year-old female was admitted with a history of an orthotopic heart transplant at 8 years of age for dilated cardiomyopathy, followed 11 years later by a diagnosis of mixed phenotype acute leukemia (including both B-cell acute lymphocytic leukemia and acute myeloid leukemia). She was admitted for chemotherapy and was cachectic on admission. After a week in the hospital, she developed MODS and extreme hyperferritinemia (21,378 ng/mL; Figure 1(d)) and an elevated IL-6 (1,040.8 pg/mL); she also developed persistent pancytopenia (with both lymphocytopenia and neutropenia). She received her first dose of tocilizumab on HD 8, and the next day, the IL-6 remained elevated (1,060 pg/mL) and the first peak in ferritin (25,108 ng/mL) was noted. CSS was then diagnosed, and she was treated with tocilizumab. MODS resolved a week later.

On HD 13, she was fungemic with *Candida parapsilosis* and several days later with *Candida krusei*, both of which were refractory to treatment; several days later, she was also bacteremic with *Staphylococcus epidermidis*. On HD 35, extreme hyperferritinemia recurred, followed by recurrent MODS associated with CSS. On HD 53, MODS worsened, and extreme hyperferritinemia was noted a third time on HD 54; she was treated with tocilizumab a second time.

On HD 67, both IL-2 and IL-6 were elevated (1,060 and 12,376 pg/mL, respectively; the last dose of tocilizumab was 12 days earlier) and the following day extreme hyperferritinemia (31,085 ng/mL) was noted a fourth time. She received a third dose of tocilizumab on HD 69 but died the following day with sepsis and refractory leukemia.

There was no autopsy for this patient.

In summary, 3 of the 4 episodes of extreme hyperferritinemia in this patient were associated with the onset or worsening of MODS. There were too few paired values of IL-6 and ferritin to investigate an association. There was no association between the trends in ferritin and CRP (*p*=0.08). The trend in ferritin correlated with the trend in D-dimer (*p*=0.005), though the number of d-dimer values was limited (*n* = 9) and only available from the first month of hospitalization, and the peak in d-dimer for this patient was less than in the other patients.

## 4. Discussion

Recurrent extreme hyperferritinemia was noted in this case series of critically ill adolescents with systemic inflammation prior to their death: episodes of recurrent extreme hyperferritinemia in these patients were often associated with MODS and an elevated IL-6, and immunodeficiency and cachexia were common to all patients. In the absence of similar data, it is possible that recurrent extreme hyperferritinemia is a biomarker of an increased mortality risk in patients with a similar presentation, though additional data are needed.

As ferritin functions both as a cellular iron storage protein and as an acute phase reactant, the differential diagnosis of hyperferritinemia is broad; however, extreme hyperferritinemia in pediatric patients has only been reported with HLH [3], though this case series suggests that a closely related systemic inflammatory syndrome—CSS—may also be associated. In addition, extreme hyperferritinemia may be associated with adult-onset Still's disease, and death associated with that disorder has been noted in a young adult [9]. Whether extreme hyperferritinemia contributes directly to inflammation, or is a marker of inflammation, or is a protective response to inflammation or whether ferritin in each patient is a result of several of these processes remains incompletely understood [10]. In any case, an animal model suggests the importance of macrophage secretion of ferritin during inflammation [11].

The phenotype of patients in this case series included persistent inflammation, immunosuppression, and cachexia, consistent with PICS [4], in which an exaggerated response to inflammation may lead to muscle breakdown and progressive organ dysfunction, as well as infections associated with immunosuppression. Proinflammatory cytokines like IL-6 have been implicated in this syndrome [12], and PICS has been associated with an increased mortality risk in critically ill adults [13].

The association of peripheral blood ANC and ferritin in the only patient without neutropenia suggested a possible PICS-related pathophysiology: chronic inflammation associated with critical illness may lead to “aberrant myelopoiesis” [4], as the bone marrow responds to tissue injury by the continued production and release of neutrophils, leading eventually to lymphocytopenia [4]. The other 3 patients in this series also had lymphocytopenia, but unrelated to “aberrant myelopoiesis.” In any case, all patients in this series had evidence of suppressed adaptive immunity, and all had potentially life-threatening infections during their critical illness.

Improved outcomes in similar patients will depend on a better understanding of risk factors and underlying pathophysiology, and biomarkers may help inform therapy. An example might be COVID-19-associated hyperferritinemia associated with increased IL-6, in which ferritin and IL-6 may serve as biomarkers of ferroptosis: ferroptosis inhibitors may have a therapeutic role in that setting [14]. It is possible that additional biomarkers, like CRP, d-dimer, or the peripheral blood ANC, may help further define the pathophysiology of inflammation in subsets of patients.

Data identifying ferritin, IL-6, and other potential biomarkers in this retrospective case series are limited by the clinical testing strategy. Any statistical associations between the trends in ferritin and these biomarkers are also, of course, dependent on the testing strategy, and there was no systematic approach to testing. Additional data on IL-6, CRP, and d-dimer might have helped affirm or reject an association with extreme hyperferritinemia in these patients. In addition, as HLH in adults is associated with elevated levels of soluble IL-2 receptor [15], as noted during episodes of extreme hyperferritinemia in 2 of the patients in this study, additional levels of this biomarker might also be helpful.

Another potential limitation to this study is that the definitions of systemic inflammatory disorders like CSS and HLH may lead to diagnostic overlap and clinical uncertainty. Limitations to this study also include the (mostly) observational nature of PICS, as well as the microbiologic complexity of inflammation in critical illness. Multiple microbiologic mediator cascades are commonly triggered during inflammation, and interactions between various mediators remain incompletely understood.

In any case, recurrent extreme hyperferritinemia—often with MODS—was noted in 4 adolescents with systemic inflammation and elevated IL-6 who did not survive their hospitalization. Additional data may help determine if recurrent extreme hyperferritinemia is a novel biomarker of increased mortality in this setting, possibly in association with PICS.

## Figures and Tables

**Table 1:** Epidemiologic data for 4 critically ill adolescents with recurrent extreme hyperferritinemia.

	Patient #1	Patient #2	Patient #3	Patient #4
Age (years)	15	16	16	19
Gender	Female	Male	Male	Female
Underlying disease	Aplastic anemia	Crohn's disease, histiocytic sarcoma	X-linked inhibitor of apoptosis deficiency	Mixed phenotype acute leukemia
COVID-19 infection	Yes	No	Yes	No
HSCT	Yes	No	Yes	No
Inflammation	CSS	HLH	HLH	CSS
Immunosuppression	Lymphocytopenia and neutropenia	Lymphocytopenia	Lymphocytopenia and neutropenia	Lymphocytopenia and neutropenia
Nutritional state	Cachexia	Cachexia	Cachexia	Cachexia
First peak in ferritin (ng/mL)/HD	11,856/79	16,809/3	30,164/15	25,108/9
Last peak in ferritin (ng/mL)/HD	22,360/175	36,617/22	>100,000/^*∗*^	36,241/69
Peak in IL-6 (pg/mL)/HD	1,423/178	>4,200/^*∗*^	36,115/99	12,376/67
Peak in CRP (mg/L)/HD	120/97	117/1	NA	>300/^*∗*^
Peak in D-dimer (microg/mL)/HD	11.31/114	17.48/4	>20/^*∗*^	3.77/16

^*∗*^The peak for this biomarker was noted on several different days for this patient. CRP: C-reactive protein, CSS: cytokine storm syndrome, HLH: hemophagocytic lymphohistiocytosis, HD: hospital day, HSCT: hematopoietic stem cell transplant, and NA: not available.

## Data Availability

Data access is restricted due to patient privacy concerns at the institution.
